# Supplementary material for: An On-Demand pH-Sensitive Nanocluster for Cancer Treatment by Combining Photothermal Therapy and Chemotherapy
Source: Pharmaceutics. 2020 Sep 2;12(9):839. doi: 10.3390/pharmaceutics12090839 (PMC7558381; doi:10.3390/pharmaceutics12090839)
Supplement: Supplementary file 1 [file pharmaceutics-12-00839-s001.pdf]

pharmaceutics

MDPI

Supplementary Material: An On-Demand pH-Sensitive Nanocluster for Cancer Treatment by Combining Photothermal Therapy and Chemotherap

Taehoon Sim, Chaemin Lim, Ngoc Ha Hoang, Yuseon Shin, Jae Chang Kim, June Yong Park, Jaewon Her, Eun Seong Lee, Yu Seok Youn, and Kyung Taek Oh

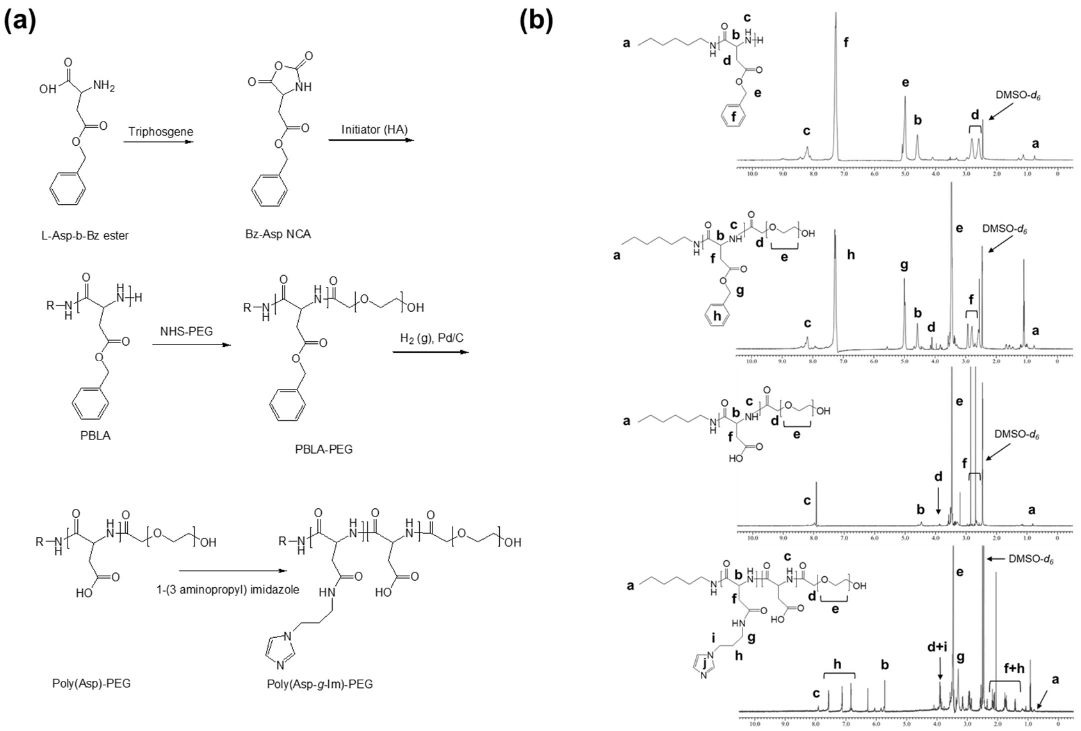

Figure S1. Chemical synthesis route and structural analysis of Poly(Asp-*g*-Im)-PEG (PAIM-PEG) by ^{1}H-NMR analysis.

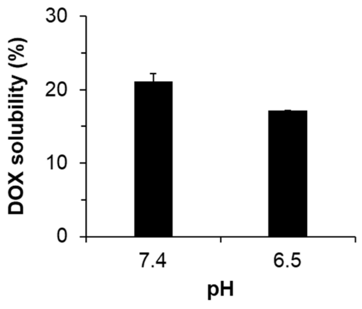

Figure S2. Solubility of Doxorubicin (DOX) in PBS at pH 7.4 and 6.5.

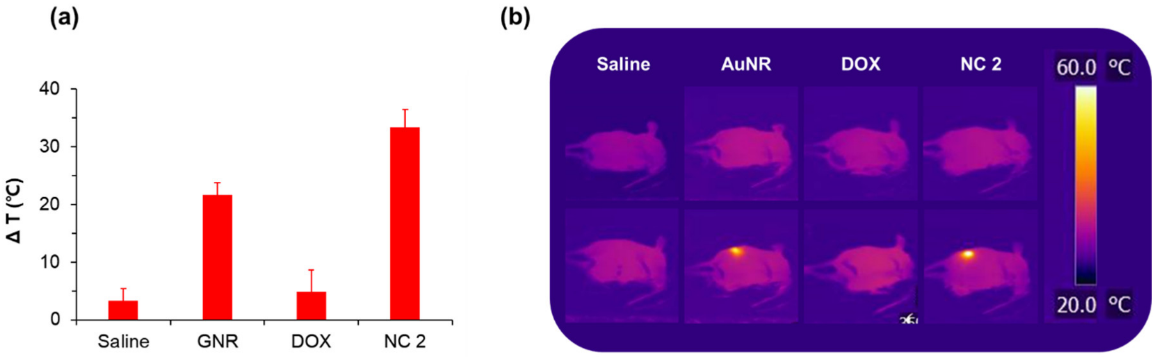

Figure S3. in vivo photothermal effect at the tumor site by the irradiation of NIR laser (808 nm, 1 min, 2 W/cm^2).
